# Editorial

**Published:** 1870-08

**Authors:** 


### Editorial.

***The American System.***

Our vivacious contemporary, the New York *Medical Gazette*, takes seriously to heart the lecture read him about his naughty *facetiæ*, by the venerable Mentor of the late Editorial convention. Incontinently repenting, he labors forthwith to bring forth "fruits meet for repentance." But the difficulty with him is ingrained, and although we have sometimes been saddened by his voluntary facetiousness, we are oppressed by inextinguishable laughter at his temporary excursion into, for him, "fresh fields and pastures new" of oracular solemnities of discourse.

The *Gazette* says that "the medical press of Great Britain and France is unanimous in urging the immediate reform of schemes of medical education which there are already as far superior to ours as Hyperion to a Satyr." Not only this, but he would almost agree to maintain contented silence for the rest of his lifetime if he could see as high a point attained here as that from which our British brethren start in their measures for further reform.

The remedy proposed by our newly reformed facetious contemporary, is in duplicate—one or two voluntarily endowed colleges so as to be nearly or quite independent of fees, thus enabled to look for quality rather than quantity, and to make their preliminary requirements as strict as some of them would now if they could; when there shall be one standard of examinations irrespective of the teaching corporations. Our contemporary is kind enough to admit that: "We have in our single degree a simpler and better system than the British one with its several qualifications to be obtained from different sources, and its multiplicity of titles which confuse even their *professors*." (?)

The present writer and responsible Editor of this *Journal*, respectfully propounds the following opinions, not begotten of the hot fancies of youth, but the product of matured conviction after a quarter of a century's experience as a medical teacher and general observer of the *status* of the profession:

1. The AMERICAN SYSTEM is superior to the British, French, Prussian or Chinese systems.

2. The right answers to three questions are superior in merit to the *required* wrong answers to forty.

3. That the average American student will receive and comprehend the facts brought before him in the medical college lecture room, as well as the average British, French, Prussian or Chinese student.

4. That anatomy is as thoroughly taught in the majority of American medical colleges, as in any European or Chinese school.

5. That when Prof. Huxley lamented, in a recent speech, that Physiology was taught and received in British schools "as if it were a mere matter of books and hearsay," he might have extended the remark to embrace the entire body of their teaching, whereas in American schools (at least in Rush Medical College under the care of Prof. Freer) Physiology is taught in a way in every particular as demonstrative as anatomy, and its immediate bearings upon hygiene and therapeutics as definitely illustrated as the bearings of anatomy upon surgery.

6. That chemistry in like manner is as thoroughly taught in a score of American medical colleges as in the most vaunted schools of the old world.

7. That the same is true with regard to general therapeutics and materia medica, with the addition of a wholesome scepticism in regard to much of the latter, which the absurdly "conservative" notions of the transatlantic schools will in no wise permit.

8. That the true relations of the specialties to general practice are better understood, and each less exclusively relied upon in America than elsewhere in Christendom or China.

9. That so far as anything can be determined by statistics, the application of the elementary principles to the practice of the art shows a better result, whether in medical, surgical or obstetrical practice, than the European records can produce. Subordinate to this statement we claim that the average graduate of the respectable American college will have a better chance of success in general practice, than his, average, imported competitor, however many Examining Boards have compelled him to answer absurd and unnecessary questions with equally foolish and obsolete forms of words.

10. The object of medical teaching is not merely to store the mind with facts, but to cultivate the capacity of students to think right on medical subjects. The method of Bacon is better than that of the mediæval schoolmen. Sound judgment is infinitely preferable to mere memory. The best medical teacher is not the one who can best cram his pupils with notions, but he who can elevate them to a high plane of medical thought. There are many learned men, but few wise. There are many whose only approach to wisdom is that they cry aloud in the streets (or medical conventions) and no man regards them.

11. There are two modes of competition between the medical colleges of this country, and the profession understand them. There is the competition of fees, and the competition of merit. The public do not understand the difference. Not one graduate in a hundred is asked where he got his diploma. The American cares little for parchments of any sort—he judges by results. If he finds an intelligent, clear-headed man he is quite apt to trust him even with his health and life. If the individual is dull and thick-headed, he will sooner try a homœopathist or a woman.

When the graduates of a college succeed, that college will have students whatever its fees, or whether its faculty make clamorous speeches in conventions or not. In America, at least, "nothing succeeds like success."

12. Sound opinions will secure their own adoption without incessant talking about them, and therefore we shall have no time to spare for further controversy on American Medical Education.

### *Underbidding.*

At the late Medical Teachers' Convention, Prof. A. Hammer offered the following resolution, seconded by Prof. Moore:

*Resolved*, That the attempt on the part of any existing schools, to reduce their tuition fees, or of any new College to be started, to underbid its neighbors, shall be by this body considered and pronounced to be an act of dishonesty.

After some discussion, participated in by Professors Hammer, Murphy, and Davis, the mover, with the consent of the house, withdrew the resolution.

The "entangling alliances" so long sought by the "uneasy gentlemen," we take it, may now be considered as self-dissolved. It is generally understood that the failure to adopt the above resolution was secured by certain well known agitators who contemplated a grand *coup d'etat* for approximately "free medical education." The proposed *coup*, however, has been temporarily postponed, as it is believed the same result can be practically secured by donating, or discounting, the nominal fees in a sufficient number of cases to warrant continued publication of annual catalogues.

Meanwhile, we repeat, the men who most of all have clamored about medical reform, are the very ones who most of all personally need it. Their clamor is a feeble imitation of the "stop-thief" cry of the streets. The best method of acquiring respect is to be respectable, and the best method of elevating the standard of medical education is for each medical instructor to see to it that he himself acquires all possible knowledge of the subject which he is appointed to teach. This cannot be accomplished by society resolutions.

### *Muriatic Acid in Fever.*

This is not a "new practice" or "German practice," as a correspondent surmises. It was employed by Prof. Nathan Smith as early as 1798. Of course it became more popular after having gone to Europe and returned. It is usually the case that the improvements which the active American mind develops are not received by the book manufacturers, or a certain class of flunkey devotees of transatlantic dotage, until they have passed the salt water at least twice. It is safe to say that the majority of approved methods of modern therapeutics are of American and not of European origin. It is time to frown down and laugh down the slavish deference to foreign *dicta*, both in modes of education and practice, which belittle many of our writers.

It is fair to state that Prof. Smith did not look upon the muriatic acid as in anywise a specific for typhus, but only as a subsidiary remedy, and in fact this is its appropriate position. All so-called medicines in typhoid fevers are only curative in so far as they promote on the one hand elimination and on the other repair. We do not even except veratrum viride. Control of sundry symptoms is not by any necessity of the case curative action. That is homœopathic doctrine.

***Editors' Convention.***

Our acknowledgments are due for advance sheets of proceedings of the recent convention of several of the medical editors of the country. We receive with suitable humility the apostolic lecture on how to conduct a medical journal, and shall continue as heretofore to follow him, as of old time—

*—dextræ se parvus Iülus*
*Implicuit, sequitur que patrem non passibus æquis.*
*——Ferimur per opaca locorum.*

But, positively, as will be seen by consulting previous pages of this number of the *Journal*, the pressure of original matter is just now so great that we are lugubriously fain to treasure up, as best we may, its profound teachings and leave the lecture itself to that immortality it will most deservedly and certainly achieve without our insignificant assistance. "Can a man gild refined gold, etc., etc.?" It is our earnest prayer that the medical editors at the next convention may have an equally eloquent, instructive, persuasive and entertaining "feast of reason and flow of soul," all compact.

